# Demographics, clinical features, and prognosis of rare lymphoepithelioma-like carcinoma across different anatomic sites

**DOI:** 10.1186/s43046-021-00103-2

**Published:** 2022-02-01

**Authors:** Xiaoqian Zhai, Jiewei Liu, Donghao Lu, Qinghua Zhou

**Affiliations:** 1 Lung Cancer Center, West China Hospital, Sichuan University, No. 363, Section 3, Furong Avenue, Yongning Town, Wuhou District, Chengdu, China; 2 Department of Head and Neck Cancer, Laboratory of Molecular Diagnosis of Cancer, Clinical Research Center for Breast, West China Hospital, Sichuan University, Chengdu, China

**Keywords:** Lymphoepithelioma-like carcinoma, Clinical features, Prognosis, Origination site, Survival rate

## Abstract

**Background:**

Lymphoepithelioma-like carcinoma (LELC) is an unusual histological malignancy type. Due to the rarity of this disease, we used the Surveillance, Epidemiology, and End Results (SEER) database to investigate comprehensively and systematically the prognosis factor of LELC.

**Methods:**

We identified 2079 patients diagnosed with LELC during 1973–2015 from the SEER database. LELC was classified according to the tumor site. We analyzed the clinical characteristics and estimated the hazard ratio (HR) of overall mortality of LELC at each site.

**Results:**

The nasopharynx was the most frequent site where LELC (58%) occurred. A large percentage of nasopharyngeal and pulmonary LELC patients were of Asian descent (44.5 and 32.56%, respectively). Furthermore, the majority of LELC patients were rather young when diagnosed. However, urinary bladder LELC and digestive system LELC (mean age: 69.03 and 68.05 years, respectively) were mainly to be found in older patients. Then according to Kaplan–Meier survival analysis, we found that patients with pulmonary LELC had worse survival. After adjusting for clinical tumor characteristics, pulmonary LELC patients were at increased risk of overall mortality compared with nasopharyngeal LELC either at the localized stage (HR 3.12, 95% confidence interval [CI], 1.55–6.26. *P* < 0.01) or at the regional stage (HR 1.72, 95% CI 1.03–2.88 *P* = 0.04).

**Conclusions:**

In conclusion, we found that urinary bladder and digestive system LELCs mainly were diagnosed in old people and different from other LELCs. Pulmonary LELC patients might have a bad prognosis. The origination site may represent a predictive factor for determining survival in patients with LELC.

## Background

Lymphoepithelioma is a descriptive term used to designate an undifferentiated carcinoma originally identified in the nasopharynx region [1]. It is characterized by the presence of a markedly prominent lymphoid infiltration [2, 3]. Histologically, neoplasia with similar morphologic appearances that arise outside of the nasopharynx is referred to as lymphoepithelioma-like carcinoma (LELC), which is a rare histological cancer type. To date, LELC tumors have been described in multiple anatomical sites throughout the body, including the salivary gland [4], tonsils [5], tongue [6], lung and bronchus [7], urinary bladder [8], female genital system [9, 10] and digestive system [11].

Although LELCs at different sites histologically resemble nasopharynx lympho-epithelioma, the clinical features of LELCs appear to vary according to sites. Most salivary gland LELC cases occur exclusively in Greenland Eskimo, North American Eskimo, and Chinese patients [12, 13], whereas tonsil and tongue LELCs are prevalent among the white population. In addition, pulmonary LELC has been found to be associated with Epstein–Barr virus (EBV) infection, whereas oropharynx (tonsil and tongue) and female genital system LELCs are associated with human papillomavirus (HPV) infection [14, 15]. Oropharynx (tonsil and tongue) LELC has been associated with a high incidence of cervical nodal spread [16], whereas LELC arising from other sites does not present this property.

Due to the low prevalence of LELC, most published studies of LELC have been case reports or clinical series with a small sample size. Additionally, some published population-based LELC studies have only focused on the clinical characteristics of LELC at one specific anatomic site. Therefore, our study aimed to provide a more systematic and comprehensive analysis of the differences in clinical features and prognosis among LELC at different anatomic sites using data from the Surveillance, Epidemiology, and End Results (SEER) program.

## Methods

### Data source

The SEER program databases, which are supported by the National Cancer Institute, contain information regarding cancer incidence and survival from specific geographic areas across the USA. The present study was performed using the SEER public-access database. The duration of the study was set from 1973 to December 2015. All data were extracted using the SEER*Stat software version 7.0.4.

### Ascertainment of LELC

**Fig. 1:**
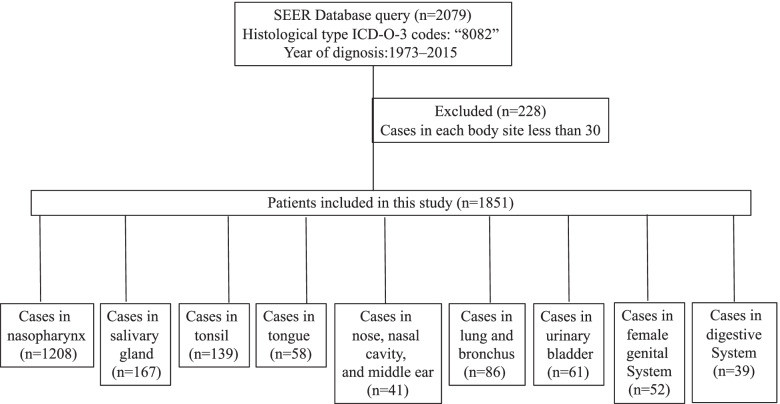
Flow chart diagram of included and excluded cases of this study

On the basis of the ICD-O-3 codes provided in the SEER database, all cases diagnosed with LELC characterized with histological tumor type “8082” were extracted. A total of 2079 LELC cases diagnosed during 1973–2015 with known patient age and tumor characteristics were identified. We categorized all LELC patients according to the tumor site. Most of the cases (1851 cases) arose from 9 sites with at least 39 cases in each site. However, the remaining 228 cases were distributed sporadically in other body parts and the number of cases in each site was less than 30. Considering the statistical significance and power, we excluded those sites in which the number of cases of LELC is less than 30. The included cases were grouped into 9 subgroups according to site: (1) nasopharynx; (2) salivary gland; (3) tonsil; (4) tongue; (5) nose, nasal cavity, and middle ear; (6) lung and bronchus; (7) urinary bladder; (8) female genital system; and (9) digestive system (Fig. 1).

Because the TNM staging system was not applicable to each patient in the SEER database, all patients were instead categorized using “SEER historic stage A”, which is a commonly used staging method in the SEER database. “SEER historic stage A” categorized tumors into localized, regional, distant, and unknown. Localized: an invasive neoplasm confined entirely to the organ of origin. Regional: a neoplasm that has extended (1) beyond the limits of the organ of origin directly into surrounding organs or tissues; (2) into regional lymph nodes by way of the lymphatic system; or (3) by a combination of extension and regional lymph nodes. Distant: a neoplasm that has spread to parts of the body remote from the primary tumor. Unfortunately, due to the small sample size of LELC patients presenting with distant disease, we were not able to conduct survival analyses for those patients.

### Statistical analysis

Basic population information and clinical features were calculated using the frequency session of SEER*Stat 14. Differences in clinical features were assessed with a chi-square test or *t*-test. The overall survival (OS) was calculated from the date of diagnosis to the date of death or the follow-up cutoff date, December 31, 2015, whichever occurred first. Patients were stratified by tumor stage for survival analysis. OS was estimated using the Kaplan–Meier method, and the log-rank test was used to assess differences between survival curves among the different anatomic sites. Multivariable survival analyses were conducted using the Cox proportional hazards model by calculating hazard ratios [HRs] and 95% confidence intervals (CIs). Year of diagnosis, age, sex, race, tumor grade, and treatment were adjusted in model 1 to estimate relative risk. All statistical analyses were performed using the software Stat 14. Differences were considered significant when *P* < 0.05.

## Results

**Fig. 2:**
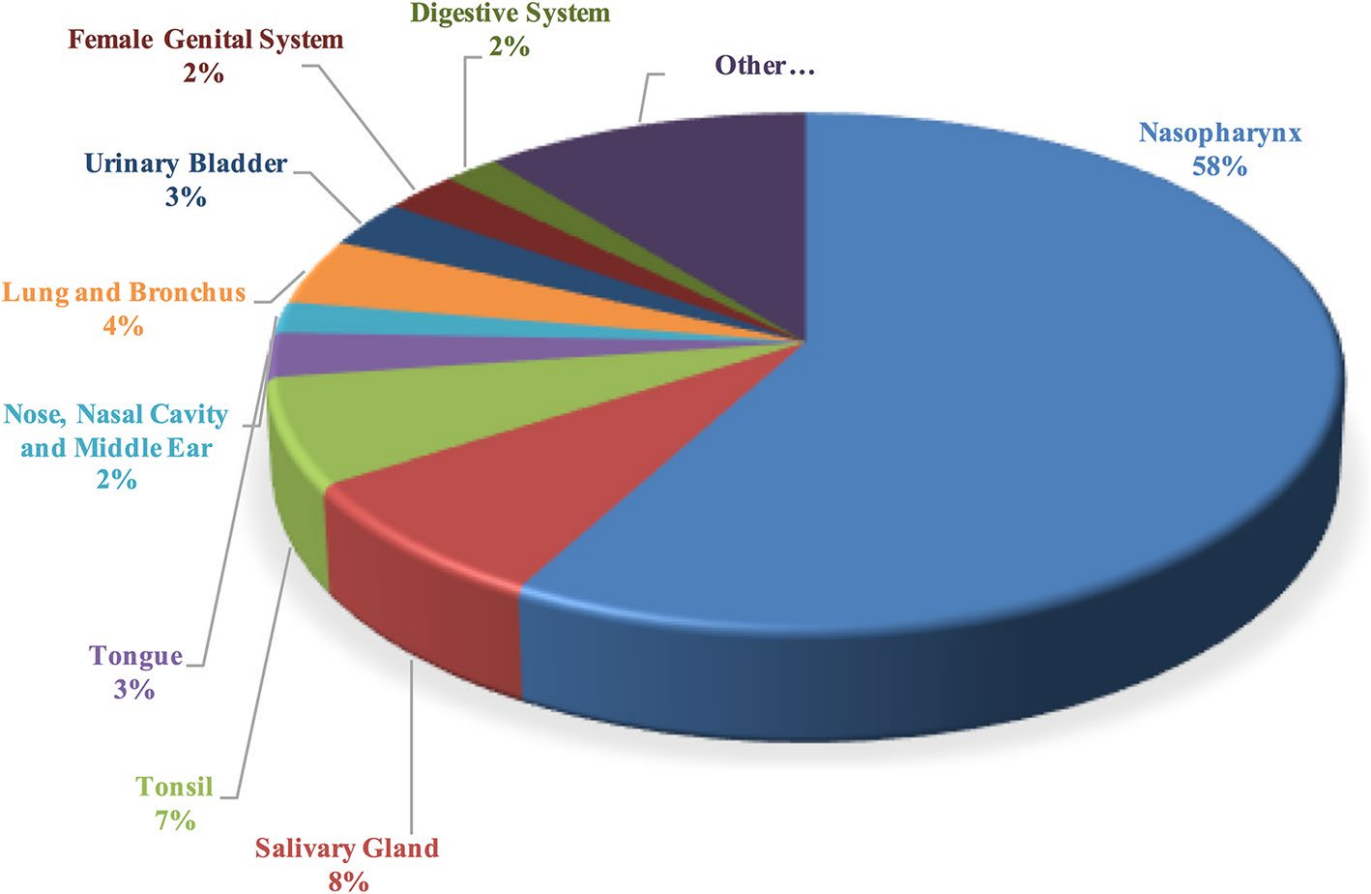
The distribution of LELC at different anatomic sites

LELC demonstrated a relatively widespread distribution: 58% of cases occurred in the nasopharynx, 8% in the salivary gland, 6.7% in the tonsil, 4.1% in the lung or bronchus, 2.9% in the urinary bladder, and 2.9% in the tongue (Fig. 2). The nasopharynx was the most frequent site associated with LELC, whereas non-nasopharyngeal LELC occurred most commonly in the salivary glands. With the exception of the brain and other components of the nervous system, LELC tumors could occur throughout the body. The majority of LELC cases occurred in patients younger than 65 years, especially cases identified in the nasopharynx (mean age: 47.6 years) and the female genital system (mean age: 49.6 years, Table 1). However, almost two-thirds of urinary bladder LELC (mean age: 69.03 years) and digestive system LELC (mean age: 68.05 years) presented in patients older than 65 years. The age for these two LELC types was significantly older than that for LELC of the nasopharynx (mean age: 47.6 years; *P* < 0.001 and *P* < 0.001, respectively). With the exception of LELC presenting in the lung and bronchus, salivary gland, and female genital system, a significantly higher proportion of LELC at other sites were associated with male cases, including 71.77% of male patients with nasopharynx LELC, 81.03% with tongue LELC, and 74.4% with digestive system LELC. In addition, large proportions of nasopharyngeal and pulmonary LELC patients were of Asian descent (44.5% and 32.56%, respectively). By contrast, LELC in the salivary gland (62.9%), tonsil (87.8%), tongue (89.6%), urinary bladder (80%), and female genital system (76.9%) were more commonly detected among white patients.

**Table 1:** Demographics and clinic features of patients with LELC according to tumor site

Site	Nasopharynx		Salivary gland		Tonsil		Tongue		Nose, nasal cavity, and middle ear		Lung and bronchus		Urinary bladder		Female genital system		Digestive system	
**Number/percentage**	**1208**	**58%**	**167**	**8%**	**139**	**7%**	**58**	**3%**	**41**	**2%**	**86**	**4%**	**61**	**3%**	**52**	**2%**	**39**	2%
**Variable**	**No.**	**%**	**No.**	**%**	**No.**	**%**	**No.**	**%**	**No.**	**%**	**No.**	**%**	**No.**	**%**	**No.**	**%**	**No.**	**%**
**Age of diagnosis**																		
Mean age	47.6		60.5		49		55		61		63.2		69		49.6		68	
≤ 65	1024	84.8	95	56.9	113	81.3	39	67.2	24	58.5	47	54.7	23	37.7	48	92.3	17	43.6
> 65	184	15.2	72	43.1	26	18.7	19	32.8	17	41.5	39	45.3	38	62.3	4	7.7	22	56.4
**Sex**																		
Male	867	71.8	79	47.3	101	72.7	47	81	28	68.3	45	52.3	41	67.2	0	0	29	74.4
Female	341	28.2	88	53.7	38	27.3	11	19	13	31.7	41	47.7	20	32.8	52	100	10	25.6
**Race**																		
White	512	42.4	105	62.9	122	87.8	52	89.6	25	61	52	60.5	52	85.3	40	76.9	31	80
Black	132	10.9	9	5.4	8	5.8	3	5.2	3	7.3	6	7	6	9.8	5	9.6	< 3	5
Asian or Pacific Islander	537	44.5	38	22.8	7	5	3	5.2	11	26.9	28	32.5	3	4.9	7	13.5	6	15
**Tumor grade**^**a**^																		
Low (1–2)	4	0.5	6	6.7	3	2.7	0	0	0	0	< 3	1.8	< 3	1.9	3	6.8	< 3	3.2
High (3–4)	802	99.5	83	93.3	109	97.3	40	100	21	100	54	98.2	53	98.1	41	93.2	30	96.8
**Tumor stage**																		
Localized	120	14.5	64	38.3	14	10.1	5	8.6	3	10.7	35	42.2	12	19.7	32	64	22	56.4
Regional	551	66.6	76	45.5	113	81.3	46	79.3	23	82.1	33	39.7	40	65.6	16	32	13	33.3
Distant	102	12.3	21	12.6	10	7.2	4	6.9	< 3	7.2	14	16.9	8	13.1	< 3	< 3	3	7.7
Unknown	55	6.6	6	3.6	< 3	1.4	3	5.2	0	0	< 3	1.2	< 3	1.6	< 3	2	< 3	2.6
**Tumor size**																		
≤ 2cm	91	23.5	35	27.1	28	36.8	16	44.4	< 3	10	26	35.1	6	15.4	19	44.2	3	8.6
2–5cm	245	63.5	78	60.5	48	63.2	18	50	7	70	36	48.7	20	51.3	14	32.5	16	45.7
> 5cm	50	13	16	12.4	0	0	< 3	5.6	< 3	20	12	16.2	13	33.3	10	23.3	16	45.7
**Nodes positive**^**b**^																		
Yes	306	33.4	65	43.1	63	55.3	21	42.9	3	13	21	25.6	8	13.1	10	19.2	9	23.7
No	610	66.6	86	56.9	51	44.7	28	57.1	20	87	61	74.4	53	86.9	42	80.8	29	76.3
**Treatment**	1208		167		139		58		41		86		61		52		39	
Surg^c^	242	20	151	90.4	85	61.2	23	39.7	15	36.6	62	72.1	57	93.4	48	92.3	31	79.5
Non-surg	966	80	16	9.6	54	38.8	35	60.3	26	63.4	24	27.9	4	6.6	4	7.7	8	20.5
Non-chemo	484	40	138	82.6	65	46.8	33	56.9	23	56	48	55.8	29	47.5	36	69.2	28	71.8
Chemo	724	60	29	17.4	74	53.2	25	43.1	18	44	38	44.2	32	52.5	16	30.8	11	28.2
XRT	1078	89.2	113	67.7	117	84.2	51	87.9	33	80.5	20	23.3	5	8.2	22	42.3	10	25.6
Non-XRT	130	10.8	54	32.3	22	15.8	7	12.1	8	19.5	66	76.7	56	91.8	30	57.7	29	74.4

*Abbreviations*: *Surg* surgery, *XRT* radiation therapy, *Chemo* chemotherapy

^a^Grade: grade 1, well differentiated; grade 2, moderately differentiated; grade 3, poorly differentiated; grade 4, undifferentiated; anaplastic

^b^Nodes positive is only available after 1988

^c^Surgery is only available after 1998

Although information regarding tumor grade and tumor size was not available for each case, the present data still demonstrated a tendency: over 93% of LELC cases at each anatomic site were classified as high grade, and the tumor sizes of most LELC cases were 2–5 cm. In addition, nodal positivity was uncommon for any site except the tonsil (55%). Accordingly, only LELC of the tonsil was typically classified as being in the regional stage at presentation (81.3%). Metastasis was rare for LELC associated with any site. In terms of treatment, radiation rather than surgery appeared to be the first-line option for LELCs in the nasopharynx (89%), tonsil (84%), and tongue (88%), whereas surgery was preferred for LELCs in the salivary gland (90%), lung and bronchus (72%), female genital system (92%), digestive system (79%), and urinary bladder (93%). Approximately 60% of cases with non-nasopharyngeal head and neck LELCs (salivary gland, tonsil, and tongue) received both radiation and surgery. Chemotherapy was often chosen as the second-line treatment, especially for cases associated with metastasis.

**Fig. 3:**
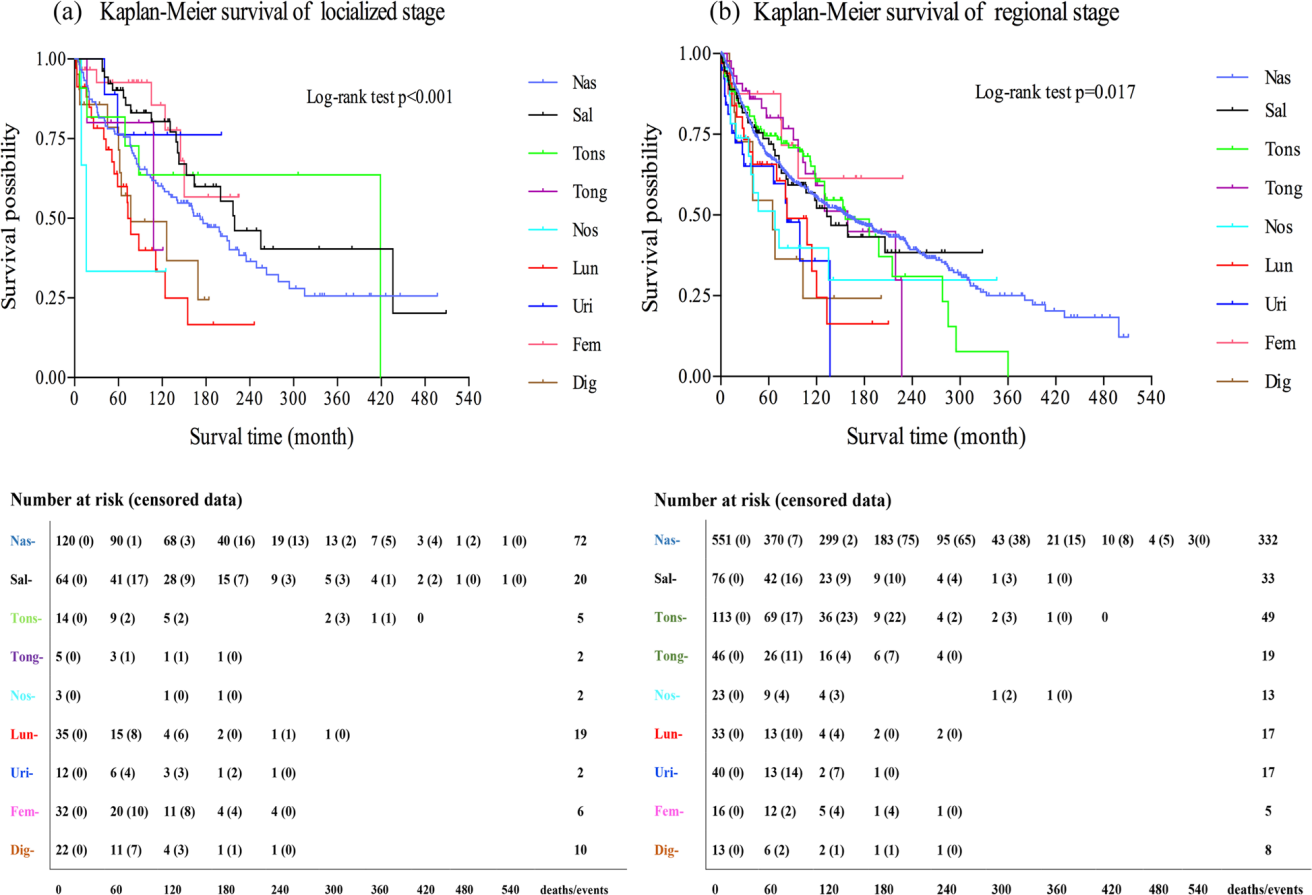
The overall survival curves of LELC patients by different anatomic sites. **a** Kaplan–Meier curves of OS and at-risk table for patients presenting with localized disease, by anatomic site. **b** Kaplan–Meier curves of OS and at-risk table for patients presenting with regional disease, by anatomic site. Nas, nasopharynx; Sal, salivary gland; Tons, tonsil; Tong, tongue; Nos, nose, nasal cavity and middle ear; Lun, lung and bronchus; Uri, urinary bladder; Fem, female genital system; Dig, digestive system

Because stage is such an important predictor of prognosis, we stratified the tumors using the SEER Historic Stage A system (localized and regional stage) to perform survival analysis. Figure 3 a and b showed the Kaplan–Meier curves of OS for patients with localized and regional disease, respectively. The median follow-up duration was 75 months. The overall survival analysis demonstrated that LELC arising from distinct sites had different survival rates (Fig. 3). We found that the median survival of localized pulmonary LELC patients was 73 months [95% CI, 51–124 months]. Further, we performed pairwise comparisons of localized and regional LELC survival in the nine sites, respectively. Localized LELC arising from the lung or bronchus was associated with worse survival compared with LELC of the nasopharynx, urinary bladder, salivary gland, and female genital system (log-rank *P* < 0.01, *P =* 0.03, *P* < 0.01 and *P* < 0.01, respectively; Table 2). Additionally, LELC of the salivary gland or female genital system was associated with better survival compared with LELC of the digestive system (log-rank, *P* < 0.01 and *P* < 0.01, respectively; Table 2). Among patients with regional disease at diagnosis, we were trying to study the difference of OS between LELC presenting in the lung or bronchus, nasopharynx, tonsil, and tongue. We found that pulmonary LELC was inferior to others (log-rank, *P* = 0.04, *P* = 0.02 *P <* 0.01; Table 3). Meanwhile, the survival rates for patients with LELC of the urinary bladder were worse than those for LELC of the nasopharynx, tonsil, and tongue (log-rank, *P* < 0.01, *P* < 0.01, and *P* = 0.01, respectively; Table 3). However, the survival rate of patients with LELC of the tonsil or tongue was significantly better than that for LELC of the digestive system (log-rank, *P* = 0.043 and *P =* 0.02, respectively, Table 3).

**Table 2:** Pairwise comparisons of localized LELC survival in the nine sites

***P*** value*	Nas	Sal	Tons	Tong	Nos	Lun	Uri	Fem	Dig
**Nas**		0.84	0.6	0.84	0.11	< 0.01	0.27	0.08	0.12
**Sal**			0.6	0.15	< 0.01	< 0.01	0.94	0.29	< 0.01
**Tons**				0.84	0.21	0.06	0.53	0.51	0.19
**Tong**					0.34	0.48	0.42	0.1	0.77
**Nos**						0.67	0.04	0.01	0.39
**Lun**							0.03	< 0.01	0.51
**Uri**								0.94	0.12
**Fem**									0.01

Abbreviations: *Nas* nasopharynx, *Sal* salivary gland, *Ton* tonsil, *Ton* tongue, *Nos* nose, nasal cavity and middle ear, *Lun* lung and bronchus, *Uri* urinary bladder, *Fem* female genital system, *Dig* digestive system

*P value with log-rank test

**Table 3:** Pairwise comparisons of regional LELC survival in the nine sites

***P*** value*	Nas	Sal	Tons	Tong	Nos	Lun	Uri	Fem	Dig
**Nas**		0.79	0.99	0.79	0.66	0.04	0.01	0.33	0.6
**Sal**			0.76	0.69	0.13	0.11	0.06	0.06	0.11
**Tons**				0.99	0.08	0.02	< 0.01	0.48	0.04
**Tong**					0.06	0.01	< 0.01	0.46	0.02
**Nos**						0.91	0.91	0.06	0.85
**Lun**							0.71	0.05	0.68
**Uri**								0.05	0.95
**Fem**									0.05

*Abbreviations*: *Nas* nasopharynx, *Sal* salivary gland, *Ton* tonsil, *Ton* tongue, *Nos* nose, nasal cavity and middle ear, *Lun* lung and bronchus, *Uri* urinary bladder, *Fem* female genital system, *Dig* digestive system

*P value with log-rank test

Table 4 shows that localized LELC presenting in the lung or bronchus was associated with an approximately 2-fold increase in overall mortality risk compared with localized tumors in the nasopharynx. The HRs changed slightly after adjustment for other factors (year of diagnosis, age, sex, race, grade, and treatment), with that for LELC of the lung or bronchus increasing to 3.12 (95% CI, 1.55–6.26, *P* = 0.001). In addition, localized LELC in the nasal cavity and middle ear had an HR of 5.44 (95% CI, 1.24–23.92, *P* = 0.0025) for mortality compared with the same stage LELC of the nasopharynx after adjustment. No significant difference in mortality risk was observed between LELC of the nasopharynx and any other sites.

**Table 4:** Survival analysis for patients presenting with localized disease

Site	Unadjusted model: crude hazard ratio (95% CI)	***P***	Model 1^a^: hazard ratio (95% CI)	***P***
**Nasopharynx**	1	Ref	1	Ref
**Salivary gland**	0.62 (0.38–1.03)	0.063	0.8 (0.43–1.49)	0.487
**Tonsil**	0.81 (0.33–2.01)	0.653	0.91 (0.34–2.39)	0.841
**Lung and bronchus**	2.16 (1.28–3.63)	0.004	3.12 (1.55–6.26)	0.001
**Urinary bladder**	0.51 (0.12–2.08)	0.346	0.81 (0.18–3.68)	0.789
**Female genital system**	0.5 (0.22–1.16)	0.109	1.09 (0.402–2.95)	0.867
**Digestive system**	1.82 (0.93–3.56)	0.08	1.93 (0.85–4.42)	0.118

*Abbreviations*: *CI* confidence interval, *Ref* reference group

^a^Model 1: Full model. Adjusted by year of diagnosis, age (continuous variable), sex, race, grade, and treatment

Table 5 shows that among patients presenting with regional disease, patients with lung and bronchus LELC showed a 71% increase in mortality risk (HR 1.71, 95% CI: 1.05–2.80, *P* = 0.031) compared with nasopharyngeal LELC. Meanwhile, patients with LELC of the urinary bladder showed a 96% increased risk of death (HR 1.96, 95% CI: 1.19–3.20, *P* = 0.008) compared with nasopharyngeal LELC. In the comparison between digestive system LELC and nasopharyngeal LELC, it was obvious that LELC of the digestive system was associated with a 2-fold increase in death risk (95% CI, 1.03–2.88, *P* = 0.048). After adjustment for other factors (year of diagnosis, age, sex, race, grade, and treatment), only patients with LELC of the lung and bronchus had a significantly different risk (1.72-fold increase, 95% CI, 1.03–2.88, *P* = 0.038) compared with LELC of the nasopharynx.

**Table 5:** Survival analysis for patients presenting with regional disease

Site	Unadjusted model: crude hazard ratio (95% CI)	***P***	Model 1^a^: hazard ratio (95% CI)	***P***
**Nasopharynx**	1	Ref	1	Ref
**Salivary gland**	1.07 (0.75–1.53)	0.713	1.02 (0.68–1.52)	0.937
**Tonsil**	1.01 (0.75–1.37)	0.923	1.22 (0.88–1.69)	0.233
**Lung and bronchus**	1.71 (1.05–2.80)	0.031	1.72 (1.03–2.88)	0.038
**Urinary bladder**	1.96 (1.19–3.20)	0.008	1.62 (0.95–2.76)	0.075
**Female genital system**	0.67 (0.28–1.63)	0.383	1.09 (0.439–2.73)	0.084
**Digestive system**	2.03 (1.01–4.1)	0.048	2.05 (0.99–4.23)	0.051

*Abbreviations*: *CI* confidence interval, *Ref* reference group

^a^Model 1: Full model. Adjusted by year of diagnosis, age (continuous variable), sex, race, grade, and treatment

## Discussion

LELC is a relatively rare histopathological type of malignancy. Our analysis of LELC according to differences in the site of presentation showed that LELC occurred predominantly in patients younger than 65 years, except urinary bladder and digestive system LELC (mean age: 69.03 and 68.05 years, respectively). Consistently, previous studies also have reported that patients with LELC tended to be younger, with mean ages ranging from 50 to 58 [4, 17–19]. In addition, as a study by Hasumi et al. [20] reported, 16 of 39 cervical LELC patients were younger than 40 years, and the average age of this patient population was 43.5 years. Our results showed that female genital LELC (92%) almost always presented in patients younger than 65 years. By contrast, LELC of the urinary bladder appeared in late adulthood with an average diagnostic age of 69.03 years, which was consistent with previous studies [21]. Even, an older mean age at diagnosis of 70.1 years was reported in a retrospective case series of 140 urinary bladder LELC patients [22]. Although several studies have reported that LELC in the digestive system occurred among relatively younger individuals [19, 23], this finding was not confirmed by two meta-analyses [24, 25] or in our data.

When examining differences by race, our data suggested that large percentages of nasopharyngeal and pulmonary LELC patients were of Asian descent (44.5% or 32.56%). Asia appears to be an endemic area for nasopharyngeal and pulmonary LELC. According to a review article, most cases of pulmonary LELC were identified among Asian individuals, whereas Caucasians represented a minority of cases (12%) [26]. However, among the present study cohort, because data were extracted from a western database, Caucasians with pulmonary LELC also accounted for a relatively large proportion of cases (60.47%). Moreover, we found that LELC in the western population was more likely to originate from the tonsil, tongue, and female genital system, which was consistent with several previous studies [18, 27]. Our study also found that most LELC patients were male, which was confirmed in some published studies [11, 18, 21, 22, 27]. Even, a ratio (10:3) between men and women was reported for urinary bladder LELC [21]. No sex predominance was observed among patients with LELC of the lung and bronchus or salivary gland, similar to the reports of other studies [4, 26, 28]. For tonsil and tongue LELCs, the incidence of regional lymph node involvement has been reported at approximately 70–80% [16, 18, 27]. Consistently with our study, when compared with other rare sites, LELC from the tonsil (55%), salivary gland (43%), and tongue (42.9%) presented with a high incidence of lymph node metastases. As demonstrated in our study, due to relatively high nodal positivity (55%), 81.3% of tonsil LELC cases were defined as being in the regional stage. The rarity of LELC and the resulting small sample size can influence the evaluation of age, race, or sex distributions. However, our study was based on a relatively large population, suggesting that our demographic findings may be more reliable than those of smaller studies.

In our cohort, localized LELCs in the lung and nasal cavity and the middle ear were associated with poor prognosis (Fig. 3a and Table 2). However, considering the extremely small sample (3 localized patients in Table 1), the survival data associated with LELC in the nasal cavity and middle ear during the localized stage likely have low statistical power. The Cox regression results indicated that localized LELC in the lung and bronchus was associated with a significant increase in mortality risk compared with nasopharyngeal LELCs after adjustment (year of diagnosis, age, sex, race, grade, and treatment) (Table 4). Although the prognosis of pulmonary LELC had been reported to be better than that of other pathologic types of lung cancer (such as lung adenocarcinoma) [29], it was associated with worse survival than the same pathological type of cancer arising from the nasopharynx. Furthermore, the localized stage of LELC in the salivary gland and female genital system presented with better survival outcomes than localized LELC of the digestive system (Table 2). According to the literature, salivary gland LELC did present with high 5-year and 10-year OS rates (90 and 75%, respectively) [4, 18], which was consistent with our study findings. For LELC in the regional stage at diagnosis, the mortality risk of regional lung and bronchus LELC, urinary bladder LELC, and digestive system LELC significantly increased when compared with nasopharyngeal LELC. As we described above, urinary bladder and digestive system LELCs mainly occurred in old people. Age may be a major factor affecting their prognosis. However, after adjustment for other factors (year of diagnosis, age, sex, race, grade, and treatment), only regional pulmonary LELC patients had a significantly increased risk, which indicated patients of LELC presenting in the lung might have a bad prognosis.

Defining the best treatment strategies for rare diseases is difficult. According to our study, radiation, surgery, or both was the first treatment options for LELCs at different sites. However, according to pathological assessments, LELC is characterized by prominent lymphoid infiltration in the tumor microenvironment. A relatively high level of programmed death-ligand 1 (PD-L1) was detected in a majority of pulmonary LELC cases [30]. Therefore, an immunotherapy strategy, especially checkpoint inhibitors, might play an important role in the treatment of advanced LELC patients.

## Conclusions

In conclusion, the demographic and clinical features of LELC greatly differ according to the site of origin. Urinary bladder and digestive system LELCs were different from LELCs in other sites and mainly diagnosed in old people. In addition, patients of LELC presenting in the lung might have a bad prognosis. The origination site may represent a predictive factor for determining survival in patients with LELC.

## Data Availability

Our data were retrieved from the public SEER database (https://seer.cancer.gov/) and were analyzed using the SEER*Stat software. The datasets generated or analyzed during the current study are also available from the corresponding author on reasonable request.

## Abbreviations

LELC: Lymphoepithelioma-like carcinoma
HR: Hazard ratio
SEER: Surveillance, Epidemiology, and End Results
EBV: Epstein–Barr virus
HPV: Human papillomavirus
OS: Overall survival
CI: Confidence intervals
